# Three-Body Collisions Driving the Ion–Molecule Reaction C_2_^–^ + H_2_ at Low Temperatures

**DOI:** 10.1021/acs.jpca.3c01402

**Published:** 2023-06-02

**Authors:** Christine Lochmann, Markus Nötzold, Robert Wild, Mauro Satta, Ersin Yurtsever, Francesco A. Gianturco, Roland Wester

**Affiliations:** † Institut für Ionenphysik und Angewandte Physik, Universität Innsbruck, 6020 Innsbruck, Austria; ‡ CNR-ISMN and Department of Chemistry, The University of Rome Sapienza, 00185 Rome, Italy; § Department of Chemistry, Koc University, 34450 Istanbul, Turkey

## Abstract

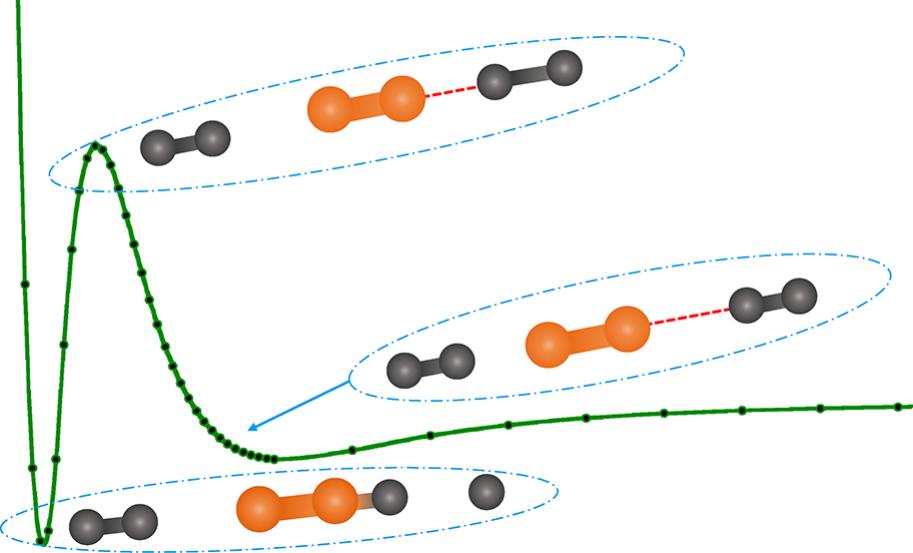

We report on the three-body reaction rate of C_2_^–^ with H_2_ producing C_2_H^–^ studied in a cryogenic 16-pole radio frequency ion trap. The reaction was measured in the temperature range from 10 to 28 K, where it was found to only take place via three-body collisions. The experimentally determined termolecular rate coefficient follows the form of  with *T*_0_ = 20 K, where *a* = 8.2(3) × 10^–30^ cm^6^/s and *b* = −0.82(12) denotes the temperature dependence. We additionally performed accurate *ab initio* calculations of the forces between the interacting partners and carried out variational transition state theory calculations, including tunneling through the barrier along the minimum energy path. We show that, while a simple classical model can generally predict the temperature dependence, the variational transition state theoretical calculations, including accurate quantum interactions, can explain the dominance of three-body effects in the molecular reaction mechanism and can reproduce the experimentally determined reaction coefficients, linking them to a temperature-dependent coupling parameter for energy dissipation within the transition complex.

## Introduction

Studying chemistry in cold environments gives insight into reaction dynamics on a fundamental level. By lowering the collision energy, quantum effects become increasingly more influential.^1,2^ For example, below 20 K, fundamental quantum processes, such as proton tunneling in the reaction D^–^ + H_2_, can be observed.^3^ Lowering the collision temperature even further (<1 mK) allows access to a regime where quantum mechanics becomes the driving force in interactions and the wave-like nature of matter becomes apparent by resonant scattering processes.^4−6^

Important processes in cold environments are three-body (3B) collisions involving atoms, ions, and molecules. Due to their atmospheric relevance, 3B processes involving nitrogen and oxygen were already studied a long time ago.^7−10^ Three-body recombination or ternary association of atoms is also an important loss mechanism in ultracold gases^11^ but is not yet fully understood. Observing slow ternary reaction rates requires long interaction times and high gas densities.^12^ In the case of ion-neutral reactions, particularly when molecular species are involved, multipole ion traps have proven to be an ideal tool that can help fulfill these requirements.^13−17^ In combined ion-atom traps, 3B collisions with ions can also be studied at ultracold temperatures.^18^ From the theoretical side, various attempts have been made to universally describe all-neutral and ion-atom-atom collisions on a fundamental level.^19,20^ Collisions involving molecular partners, however, significantly increase the internal degrees of freedom involved in the reaction and, as such, make it more difficult to accurately describe the observed rate constants through classical, semiclassical, or quantum chemical calculations.^19,21,22^ For these termolecular rate coefficients, the matching theory still has to rely heavily on experimental data to benchmark the calculations.

A 3B recombination collision involving a cation, for instance, can be described as a two-step process of the form:
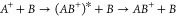
1where the intermediate complex  is stabilized by a collision with a third body *B*. In general, no bond cleavage is observed during a 3B association process for either of the collision partners. Only in a few cases has such a dissociation been observed at high collision energies and high pressures, for example, Si^+^ + C_2_H_2_^23^ and NO_2_^–^ + CO_2_.^24^

Here, we study the reaction of C_2_^–^ with H_2_, which we found to be another example where bond reactive cleavage occurs in a 3B collision process. C_2_^–^ is one of the best-characterized molecular anions. It is one of few anions with several stable electronic states and has been extensively studied.^25−28^ It has also been proposed to exist in the interstellar medium due to the abundance of its neutral counterpart C_2_, but it has not yet been identified. Hence, its chemical interaction with one of the most abundant molecules in the universe, the H_2_ partner, remains of fundamental interest as an important step for modeling its chemical evolution within interstellar media.

The reaction of C_2_^–^ with H_2_ may proceed via several possible reaction pathways, all of which are exoergic. It can either happen via two-body (2B) associative detachment of the form:
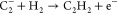
2or following a reactive collision forming a charged molecular product and occurring via either a bimolecular hydrogen transfer:
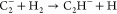
3or a 3B collision event. The latter leads to a chemical reaction according to the following step:
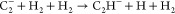
4

The reaction of C_2_^–^ with H_2_ was previously studied by Endres et al.^29^ at hydrogen densities of about 10^12^ cm^–3^ to 10^13^ cm^–3^. No charged products were detected. A 2B process following reaction 2 was assumed, and a very small rate coefficient of around 3 × 10^–16^ cm^3^/s was extracted from the data. However, the occurrence of a 3B reaction mechanism could not be ruled out.

Here, we report on the temperature-dependent 3B reaction rate coefficients for C_2_^–^ reacting with H_2_ in the temperature regime from 10 to 28 K measured in a multipole radio frequency ion trap. Additionally, we provide a comparison to a statistical 3B recombination formula, which can reproduce the temperature dependence, and to variational transition state theoretical (VTST) calculations using *ab initio* interaction forces. The VTST calculations are found to accurately provide the transition pathways between the reactants and products and the final reaction rate coefficients, in agreement with the experiments.

In the following section, we will first present a subsection on the Experimental Methods as well as a subsection with details on the Theoretical Modeling. In the third section, the results of the experiment and the comparison to the calculations are presented, followed by the Conclusions section.

## Methods

### Experimental Methods

The experiments were carried out using a 16-pole radio frequency ion trap, which is temperature variable between 300 and 6 K and combines long ion storage times with the high gas densities required to study slow 3B reactions. The ion trap can store ions with lifetimes of many hours. This, combined with buffer-gas cooling, makes it a perfect tool for probing cold chemistry.^30^ A detailed description of the setup can be found elsewhere,^28,31^ here, we will summarize the most important aspects.

The C_2_^–^ ions are created in a plasma discharge source by using a mixture of acetylene (5%), carbon dioxide (5%), and argon. The ions are then extracted toward the trapping region through a Wiley–McLaren-type time-of-flight mass spectrometer. Once trapped, they are cooled to cryogenic temperatures through buffer-gas cooling with helium. The helium thermalizes with the trap temperature by collisions with the trap walls. The ions, in turn, will cool down to the temperature of the buffer gas through elastic and inelastic collisions. The ions are then exposed to H_2_ gas for varying amounts of time at H_2_ densities between 10^12^ cm^–3^ and 10^14^ cm^–3^. The hydrogen pressure is monitored throughout the experiments with a gas-type independent capacitance gauge attached through a tube to the trap housing. Since this pressure gauge is only sensitive to pressures above 10^–5^ mbar, corresponding to in-trap hydrogen densities of ≈10^13^ cm^–3^, pressures below this range are measured with a Pirani gauge installed in the H_2_ inlet line, which is calibrated with a cold cathode gauge. Due to the temperature difference between the trap (cryogenic) and the pressure gauges (room temperature), the temperature difference as well as a transpiration effect are taken into account when determining the H_2_ density inside the ion trap.^32^ Due to these effects, we estimate an overall 10% systematic uncertainty on the hydrogen density for all densities above 2 × 10^13^ cm^–3^ determined directly through the capacitance gauge, and 40% for densities below 2 × 10^13^ cm^–3^. After the interaction, the ions are extracted from the trap and detected on a microchannel plate detector yielding time-of-flight dependent ion signals, which are used to determine the ion loss and growth rates from the trap. The same procedure was repeated at five different temperatures ranging from 10 to 28 K to determine the temperature dependence of the 3B reaction rate.

### Theoretical Modeling

Variational transition state theory following a microcanonical approach^33^ was applied to calculate the reaction rates of the bimolecular (2B) and termolecular (3B) recombination rates of C_2_^–^ with H_2_ according to reactions 2, 3 and 4. The calculations were performed after accurate *ab initio* computations had provided detailed knowledge on the most efficient minimum energy path (MEP) followed by the reacting partners, as we shall discuss below.

By incorporating within the microcanonical approach the dynamical couplings between internal states of the transition state (TS), we have used the ab initio potential energy surface (PES) for both the bimolecular and termolecular situations. We have further computed the tunneling probabilities for both the 2B and 3B mechanisms, following the formulation of the next subsection. The theoretical analysis of the bimolecular data indicates that the 2B mechanism leads to negligible reaction rate coefficients, while only the inclusion of a third body provided by an additional H_2_ molecular partner to the initial complex can yield rate coefficients that are in good agreement with the experimental findings. More details on the employed computational methods are provided in the following subsections.

For the second incoming H_2_ molecule, linear, nonlinear, and T-shaped approaches to the initial C_2_^–^-(H_2_) complex were considered. The computed barriers at the TS location were found to be increasingly lowered for termolecular structures in comparison with the simpler bimolecular configurations. The transit over the energy barrier was treated using the Eckart formulation of the tunneling transmission coefficients, and the actual shape of the barrier was fitted to the existing *ab initio* data for the termolecular TS formation.

#### The *Ab Initio* Treatment

The reaction potential energy curves, along several possible MEP approaches, were obtained by using both DFT with the B2PLYP basis set at the aug-cc-pvtz level of theory and the coupled cluster method with the RHF/UCCSD(T)-F12b basis set at the aug-cc-pVQZ level of theory. All degrees of freedom along the MEP were optimized, with the proper linear symmetry constraint, when applicable. Several thousand points were generated in order to closely follow the VTST approach along both linear and nonlinear approaches, finding the former to be the most efficient.^33^

**Figure 1:**
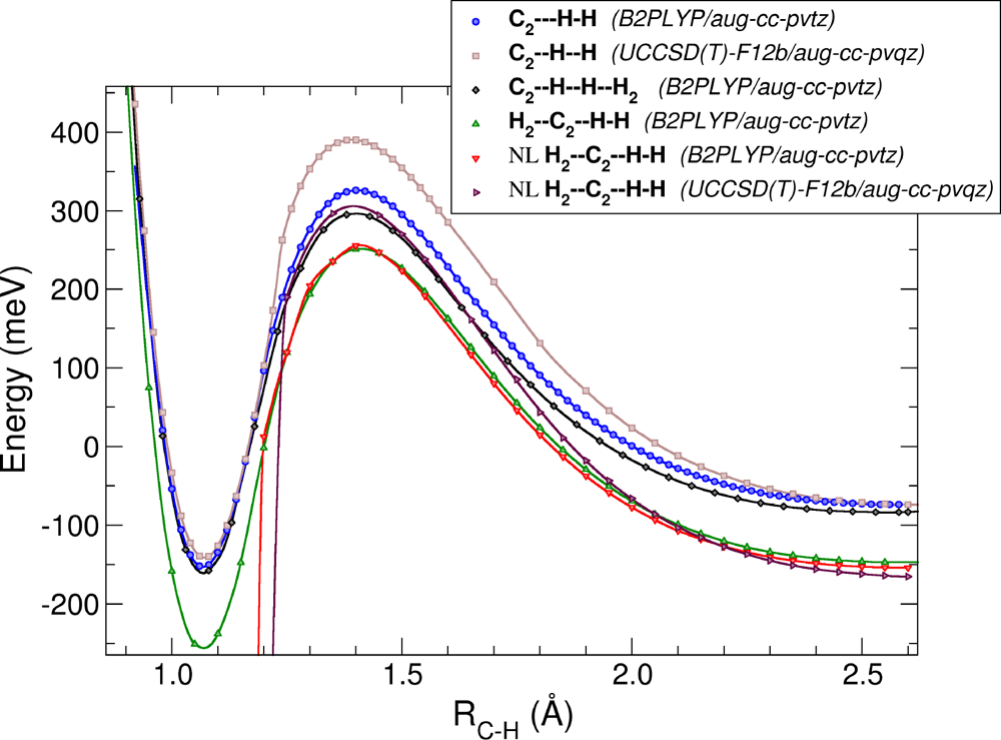
MEP energy curves along the relevant C-to-H distance, considering 2B and 3B interactions. Calculations show a lowering of the barrier when considering the 3B process, for which the green lozenge curve denotes a linear approach and the red lozenge curve the nonlinear approach. See the main text for further details.

Figure 1 shows the bi- and termolecular potential energy curves with linear and nonlinear approaching H_2_ over the reaction coordinate *R*_C–H_, calculated following the *ab initio* treatment. The nonlinear TS configurations produced much deeper wells after the barrier and, therefore, can be considered to be too stable to lead to fast reactive breakups, as is instead the case for the linear termolecular TS structures. The nonlinear structures were therefore not further treated by our kinetics model. One should notice that the discontinuity in the nonlinear MEP is due to the formation of vinylidene (H_2_C=C), an event which is not very likely to occur due to the very large amount energy produced for its formation by our calculations: 1850 meV when computed with B2PLYP/aug-cc-pvtz and of 1825 meV when computed with UCCSD(T)-F12b/aug-cc-PVQZ. Thus, when released, such a large amount of energy will eventually lead to the removal of one of the two hydrogen molecules bonded to the carbon atom, thereby discarding the 3B mechanism which is actually dominating at the low T considered here. The dynamical picture provided by our calculations is verified by the lack of experimental evidence in this study on the formation of vinylidene.

One should also notice that the different curves reported in Figure 1 have two different asymptotic energies because we have chosen the zero energy as the H_2_ + C_2_^–^ for the bimolecular MEPs and the 2H_2_ + C_2_^–^ energy for the termolecular MEPs. Hence, the termolecular MEPs end at about 75 meV lower energy at the asymptote with respect to the MEPs of the bimolecular reaction. This energy difference in the termolecular reaction channel is due to the formation of the [C_2_–H_2_]^−^ complex between the C_2_^–^ and the spectator hydrogen molecule, while the reactive H_2_ is far away. It is also the reason why the termolecular process becomes the favorite one for the present reaction at low temperatures.

**Figure 2:**
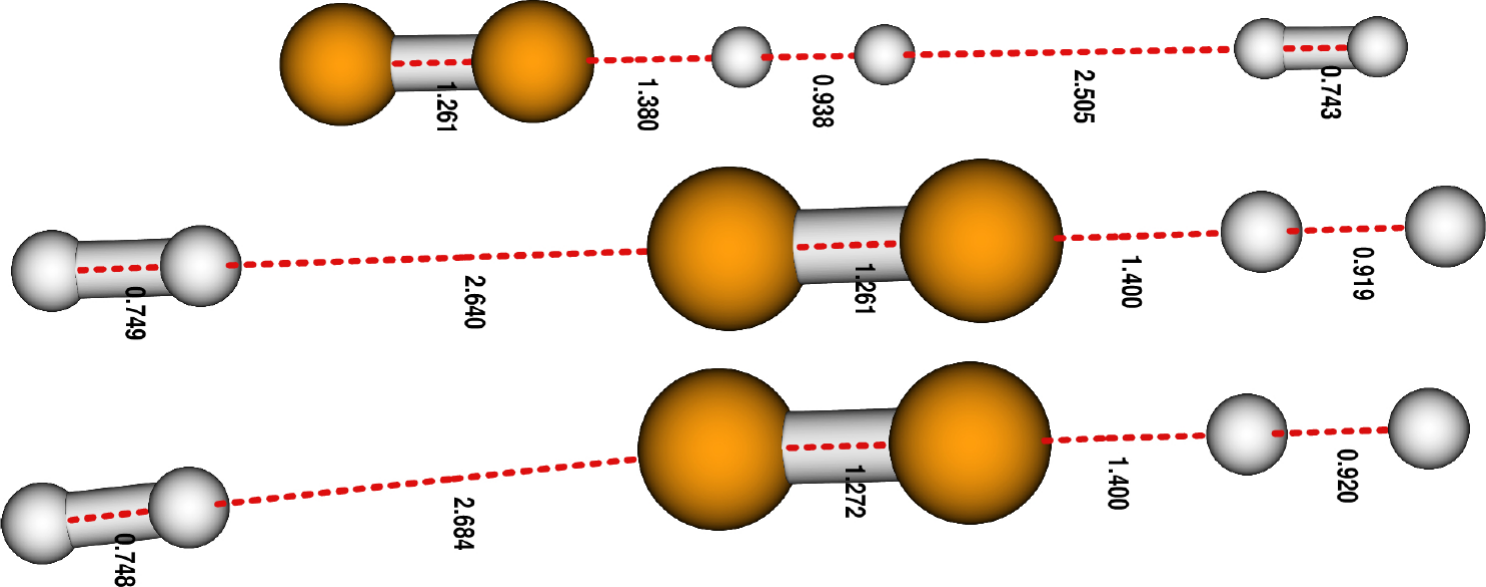
Termolecular complexes at the TS position along the MEP, which all show nearly linear configurations.

The geometries of the 3B collision complexes at the top of the barrier are shown in Figure 2. In all cases, the geometries are linear, with either both H_2_ on the same side or one on either side of the C_2_^–^. It is clear from their pictorial presentation that the present reaction strongly favors the linear approach as the most efficient MEP. Along such a path, our calculations treated all the degrees of freedom needed to describe the vibrational modes of the various TS configurations, which describe the reacting partners before, after, and on top of the barriers shown in Figure 1.

#### Tunneling and Three-Body Rate Coefficient Formulations

The tunneling transmission coefficient *K*^*tun*^(*T*) is calculated by using the familiar equation:
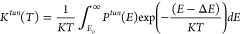
5where *E*_*o*_ is the zero energy of the reaction and *P*^*tun*^(*E*) is the tunneling probability depending on the relative kinetic energy of the reactants. It is given in the zero curvature approximation by
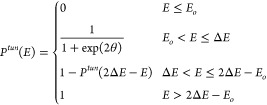
6The imaginary action integral is given by , where *V*(*s*) is the energy along the MEP defined by the coordinate *s*, and μ is the reduced mass associated with the reaction coordinate.^33−35^ The actual numerical values of the tunneling transmission coefficients are reported in Table 1. As expected, we see from the table how dramatically the tunneling efficiency changes with the temperature values, as is often found in such calculations.

**Table 1:** Calculated Tunneling Transmission Coefficients at Selected Temperature Values

Temperature (K)	Transmission coefficient
10	0.656 × 10^150^
14	0.862 × 10^106^
18	0.401 × 10^82^
24	0.226 × 10^61^
28	0.182 × 10^52^

The termolecular reaction we are considering in our present modeling is reaction 4. We have obtained the rate coefficient for this reaction by using current formulations of the VTST approach as discussed earlier, see refs (36−38). Briefly, we are starting with the adiabatic rotational approximation, which can be written as a canonical rate coefficient via the following form:
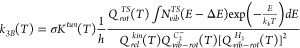
7where  and . The  and  are the rotational and vibrational molecular partition functions of the two reactants C_2_^–^ and H_2_, and *Q*_*rot*_^*TS*^(*T*) is the rotational molecular partition function at the geometry of the barrier top. Note that the quantities N_*vib*_^*TS*^ are the number of vibrational states present in the 3B complex and are calculated by direct count using the Beyer–Swinehart algorithm.^39^ The *N*_*vib*_^*TS*^ does not take into account the tunneling probability, which therefore has to be calculated separately using the specific factor *K*^*tun*^(T) given by eq 5.

For the specific reaction, our structure calculations have already indicated that the barrier height (Δ*E*) is 305.9 meV (from RHF/CCSD(T)-F12b). When the rotation-vibration couplings within the complex are actively included during their motion along the reaction MEP, then the rate coefficient expression becomes
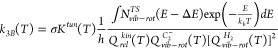
8

Eq 8 is reduced to the canonical standard rate coefficient under the ergodic hypothesis of equivalent energy partition paths as the complex proceeds along the MEP discussed earlier. If instead all accessible microstates are not equiprobable over a long period of time, then eq 8 has to take into account the unbalance in the energy distribution within the termolecular complex. This is more likely to be the case for reactions occurring at the low temperatures of the present measurements. We can then argue that the involved TS of the termolecular reaction has one of the two H_2_ molecules as the reactive species, whereas the other H_2_ is a spectator, and its binding energy with C_2_^–^ ( meV) can be partially transferred to the reactive part of the TS, as shown later in the discussion Section. This internal ‘cooling’ of the spectator molecule occurs by a transfer to the reactive coordinate of the TS of an extra amount of energy which can be evaluated as a fraction *x* of the total available energy of the H_2_ spectator, as mentioned before. Hence, the scaled rate coefficient can be rewritten as
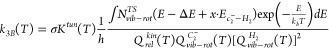
9

## Results and Discussion

**Figure 3:**
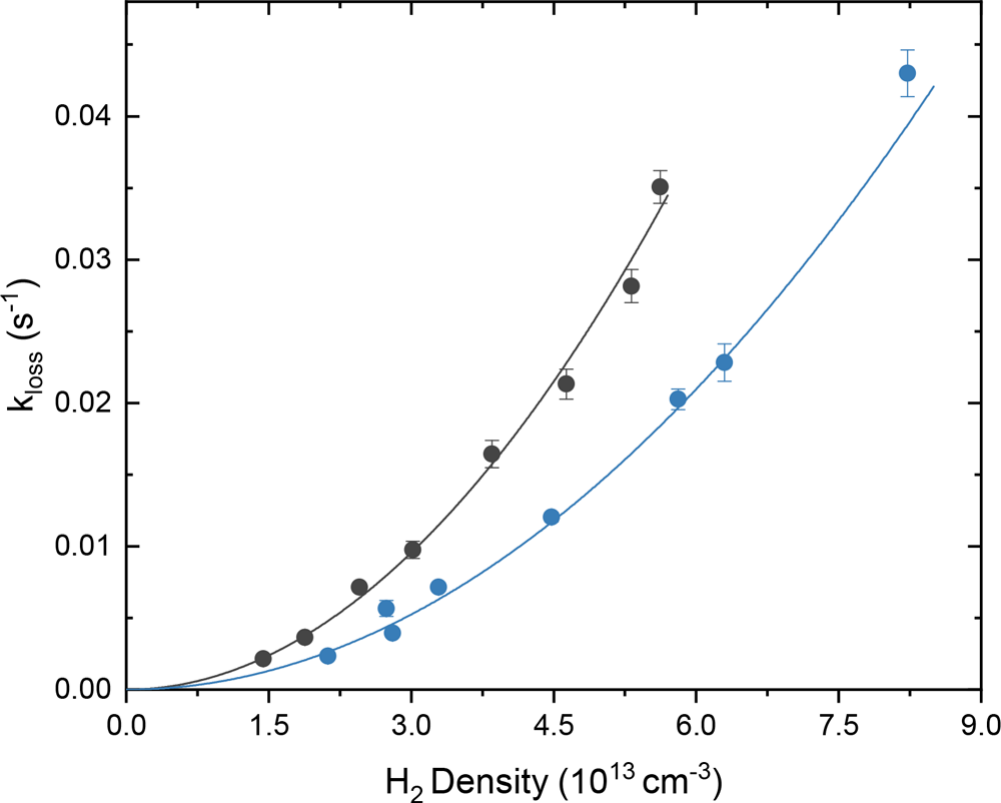
C_2_^–^ ion loss rate as a function of H_2_ density at 14 and 28 K. The solid line represents a second-order polynomial fit to the data points, which reveals a pure quadratic dependence on the density.

The temperature-dependent kinetics of C_2_^–^ reacting with H_2_ are experimentally observed via the loss rate of the C_2_^–^ parent ions and the growth rate of the C_2_H^–^ product ions as a function of interaction time in the trap at varying H_2_ densities and at five different temperatures. Besides the C_2_H^–^ product, no higher masses are detected in any of the time-of-flight spectra. Hence, clustering of H_2_ on C_2_^–^ or on C_2_H^–^ can be excluded on the time scales of these experiments. Examples of the density-dependent loss rates *k*_*loss*_ at 14 and 28 K are shown in Figure 3.

To determine the density dependence of the ion loss rate, a second-order polynomial fit is performed on the C_2_^–^ ion loss data for all five temperatures. The fit function follows the form *k*_1_ + *k*_2_*n* + *k*_3_*n*^2^, where *k*_1_, *k*_2_ and *k*_3_ are the uni-, bi- and termolecular rate coefficients, respectively, and *n* denotes the hydrogen density. The fit reveals a pure quadratic dependence on the hydrogen density, as the constant *k*_1_ and linear term *k*_2_ in the fits are consistent with zero within the 1σ error at 14 K and above. Only at 10 K trap temperature, a small contribution from a 2B reaction cannot be ruled out. This means that above 10 K, no associative detachment (reaction 2) and no bimolecular hydrogen transfer (reaction 3) events seem to occur within the experimental uncertainty, but only 3B reactions following reaction 4 are observed. Furthermore, the same analysis was performed on the C_2_H^–^ product ion growth data sets at all temperatures. The results are in good agreement with the rate coefficients determined from the ion loss data sets. However, due to the lower sensitivity in the detection of the C_2_H^–^ ion signal, this data is less reliable and was therefore omitted from the following analysis procedure.

**Figure 4:**
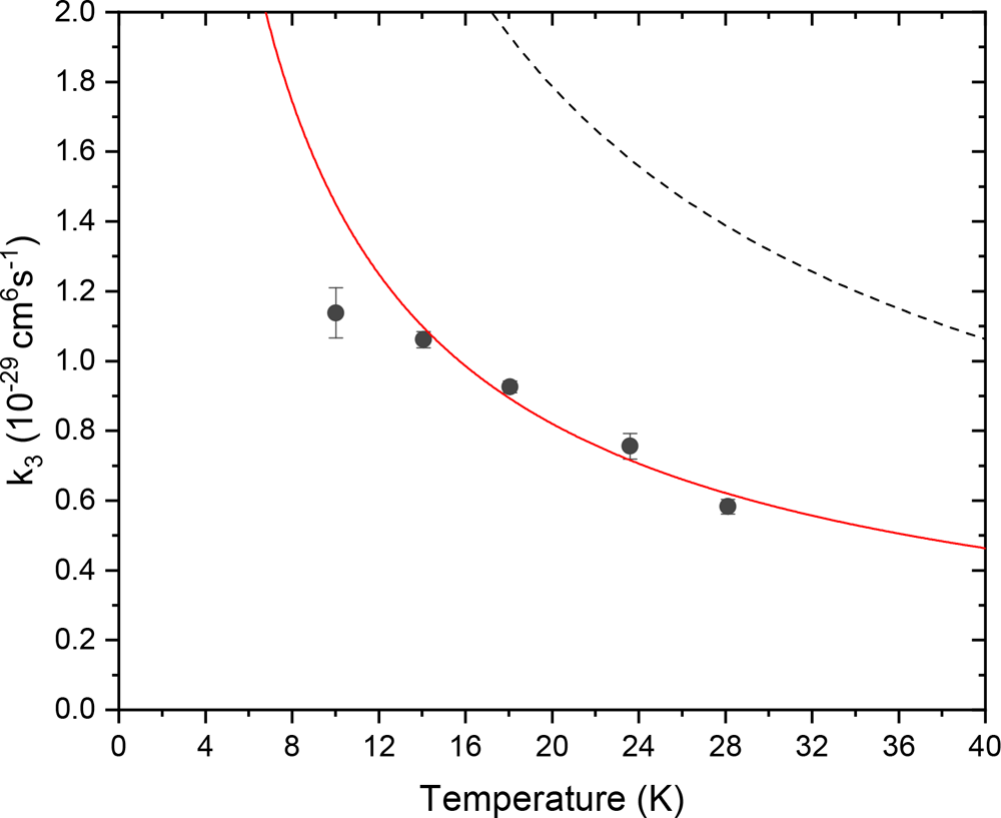
Three-body reaction rate of C_2_^–^ with H_2_ as a function of temperature. The red curve is a power law fit to the data points with the exemption of the point at 10 K. The dashed line is a comparison to the statistical ion-atom-atom 3B recombination rate derived by Perez-Rios et al.^20^

Figure 4 shows the 3B reaction rate coefficients determined from the density-dependent ion loss rate as a function of temperature. The temperature dependence of the reaction rate is obtained through a power law fit of the form  where *T*_0_ = 20 K. The 10 K point was exempted from the fit, as here the kinetic temperature of the ions might differ from the trap temperature as we previously observed in this trap for the 3B rate of Cl^–^ with H_2_.^17^ We attribute this low-temperature behavior to heating effects due to patch potentials in the trapping fields as well as heating of the ions due to micromotion on the edges of the trap, which are typical characteristics for multipole ion traps.^40−42^ As such, we cannot exclude that this point might deviate from the actual reaction rate. Additionally, 10 K is close to the freezing temperature of H_2_. Hence, the lifetime of H_2_ sticking onto the trap walls increases dramatically, introducing an extra uncertainty in the density measurement. The fit then yields *a* = 8.2(3) × 10^–30^ cm^–6^/s at *T*_0_ = 20 K and a temperature dependence of *b* = −0.82(12). This falls close to other previously reported anion-molecule 3B reaction rates. For example, at 20 K OH^–^ plus H_2_ has a rate of *k*_3_ ≃ 3 × 10^–29^cm^6^/s and Cl^–^ plus H_2_ a rate of *k*_3_ ≃ 6 × 10^–31^ cm^6^/s.^16,17^ Note, however, that for Cl^–^-(H_2_), the temperature dependence follows *T*^–1^.

Figure 4 also includes the temperature-dependent ion-atom-atom 3B recombination rate (dashed line) derived by Perez-Rios et al. within a classical approach based on hyperspherical coordinates.^20^ The temperature dependence of the experimental data is in good agreement with the *T*^–3/4^ prediction of the statistical formulation. The absolute rate coefficient of the model is larger by a factor of 2. Since the model is formulated for ion-atom-atom collisions and, as such, does not include the internal energy distribution of the involved molecules, the difference in the rate constants might be due to this important reactive mechanism, which is included in our present molecular calculations. In order to elucidate the influence of the internal degrees of freedom on the recombination rate coefficients, we performed statistical VTST calculations assuming both a 2B and 3B collision process and generating the MEP from *ab initio* quantum calculations. The structural features along the obtained MEP were in turn employed to generate rate coefficients as discussed in the preceding subsection.

Our calculations have shown that the MEP associated with a bimolecular complex provides a higher barrier height at the TS than when the termolecular complex is following the MEP for the reaction, suggesting a larger tunneling probability for the 3B process, a feature that was confirmed by our calculations. It is, therefore, instructive to view once more the actual configurations which we can depict along the MEP of the interaction potential energy.

**Figure 5:**
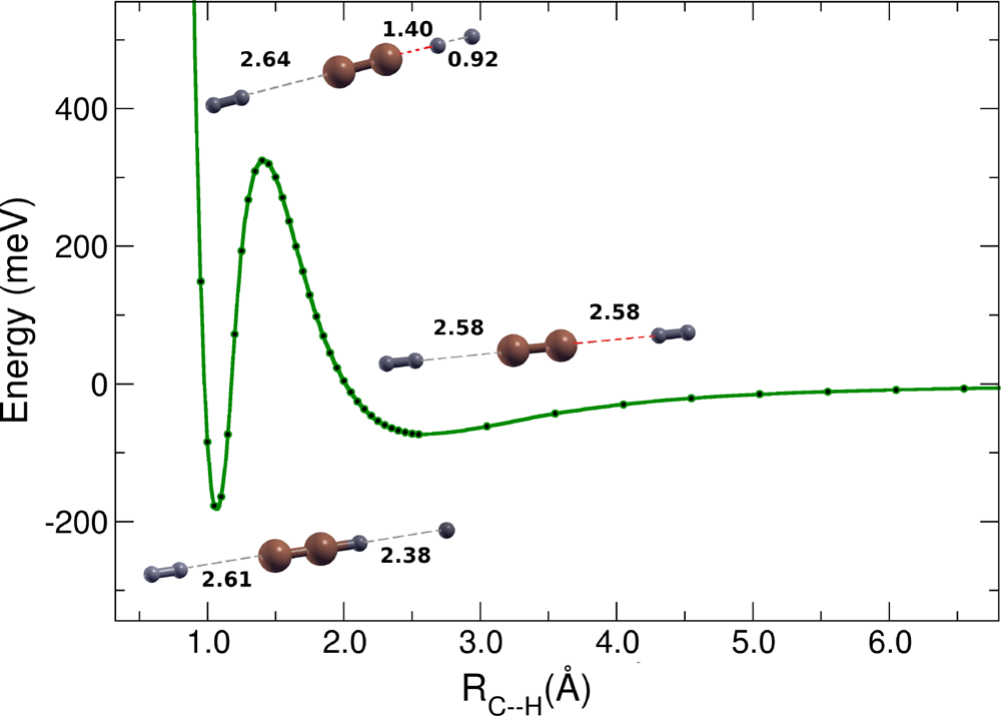
Three-body complexes depicted along the MEP and at the important locations of the chemical evolution. The numbers correspond to the bond lengths in Å within the complex. The red dashed lines show the distance of the first H_2_ partner to C_2_^–^, which decreases along the reaction coordinate *R*_C–H_. All complexes confirm the dominance of the linear configurations.

**Figure 6:**
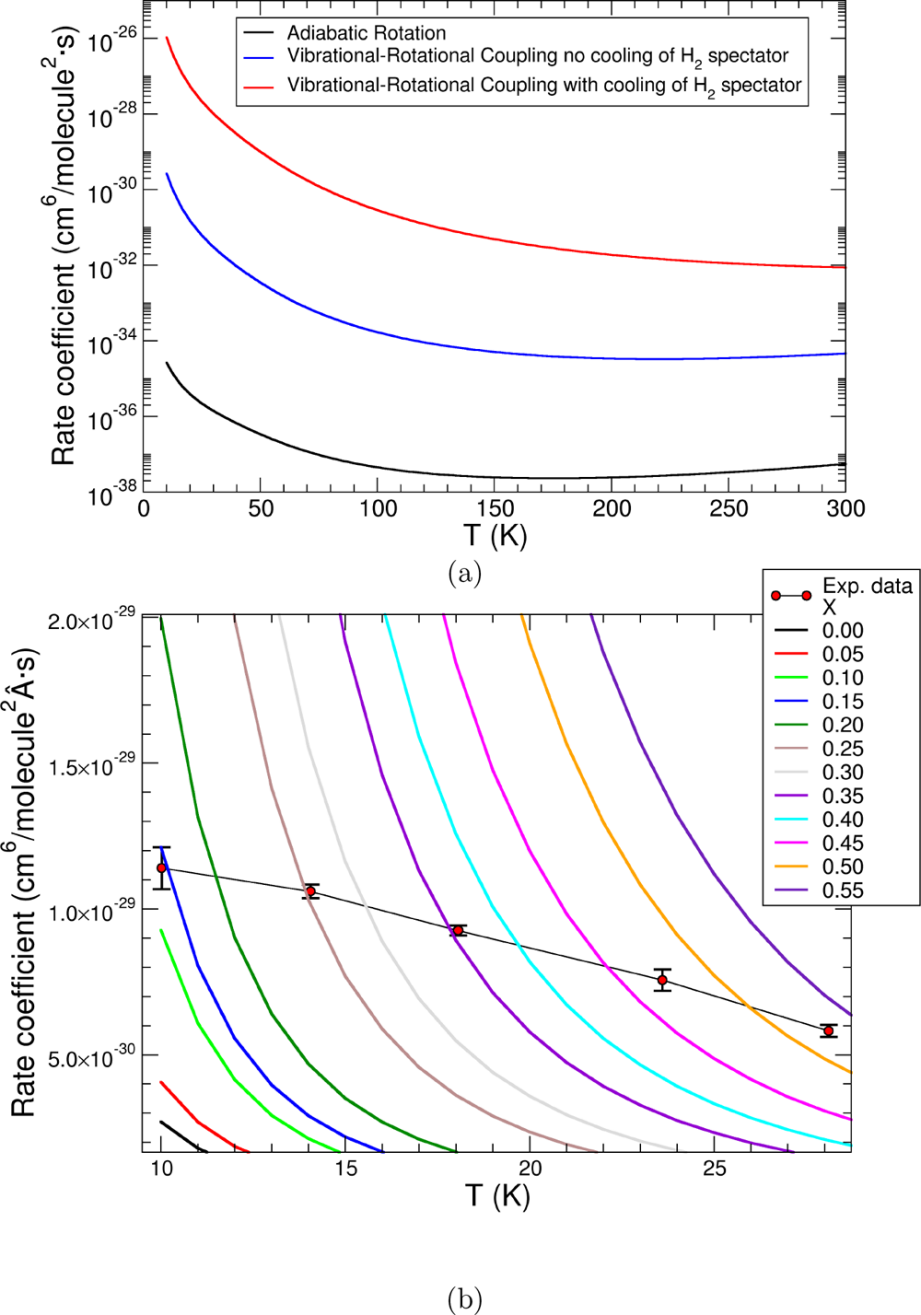
Calculated 3B recombination rate coefficients as a function of temperature. (a) Calculations are shown for different types of coupling between the complex and the bound H_2_ as well as the second spectator H_2_ which is involved in the cooling of the TS complex. (b) Amount of transferred energy from the spectator H_2_ to the complex. At higher temperatures larger fractions of energy are transferred.

We see in Figure 5 that all partners favor the collinear approach before and after the tunneling, reaching the reaction region where both the residual H atom and the spectator hydrogen molecule are about to leave. The sketch of the three partners at the top of the barrier, which is the actual TS configuration of the termolecular complex, already shows that one of the H_2_ molecules is more weakly bound to the 2B complex and will play the role of an energy depositor into the latter while on its way to the products. In our calculations, the initial complex is located on a shallow well before the barrier and can transition to the product side via tunneling through the reaction barrier, acquiring the TS configuration on its way. In the case of the termolecular process, the second H_2_ is only weakly bound to the C_2_^–^ by ≃78 meV. This is about half of the energy amount that has been gained in the shallow well on the right side of the barrier (minus the ZPE value), where the additional H_2_ molecule only acts as a chemical spectator. This energy can be partially transferred to the reactive part of the complex, and as such, the weakly bound hydrogen molecule is still involved in the redistribution of the available energy in the complex. Given the fact that the *T* values are fairly low, only the less energetic bending modes will be effectively coupled during this transfer. Ultimately this leads to the stretching of the H–H bond in the first H_2_ and to the stabilization of the C_2_H^–^ product with the loss of an H atom. Figure 6a shows the computed temperature-dependent rate coefficients in the range from 0 to 300 K obtained by following different energy coupling models within the VTST approach.

The detailed derivation of our expressions for the reaction rate coefficients (see previous section) allows us to consider the following options:

(i) an adiabatic approach, where the complex is treated as a rigid structure and no energy is exchanged between the bimolecular complex and the spectator H_2_ molecule (reported by the black curve in the upper panel of Figure 6). We clearly see that the black curve provides rate coefficients that are too small since the termolecular complex remains bound at the bottom of the well after the barrier and therefore does not break up into products fast enough;
(ii) the addition of vibrational–rotational coupling between the bound H_2_ and the C_2_^–^ within the bimolecular complex, as given by the blue curve in the upper panel of Figure 6. These new rate coefficients are seen to increase slightly but do not yet take into account the additional energetics linked to the third body, the additional H_2_ molecule;
(iii) we can further add the coupling between the complex and the second H_2_ molecule (provided by the red curve in Figure 6a). These rates include the complete transfer of the ≃78 meV of energy gained in the shallow well before the barrier. However, the full release of that amount of energy yields 3B rates that are too large in comparison with experiments.

We have shown in the previous section that one can modify this modeling and further surmise that the energy-transfer step may not be fully efficient so that only part of the available energy from the binding of the second H_2_ molecule goes to the TS, while the rest gets dispersed into other degrees of freedom not directly coupled to the reactive path. From the evaluation of such a fractional energy transfer *x* at the different temperatures, new rate coefficients can be obtained (see eq 9) and compared with the experimental data. We expect the efficiency to be *T*-dependent and therefore have created a grid of different values for the fraction *x*, mapping the range of temperature values tested in the experiments. This is shown by the data in Figure 6b. The lines correspond to the rate coefficient calculations for each value of *x* within that grid. The actual experimental values at the five temperatures studied are also superimposed on the grid to show which are the *x* values that best reproduce the experiments.

**Figure 7:**
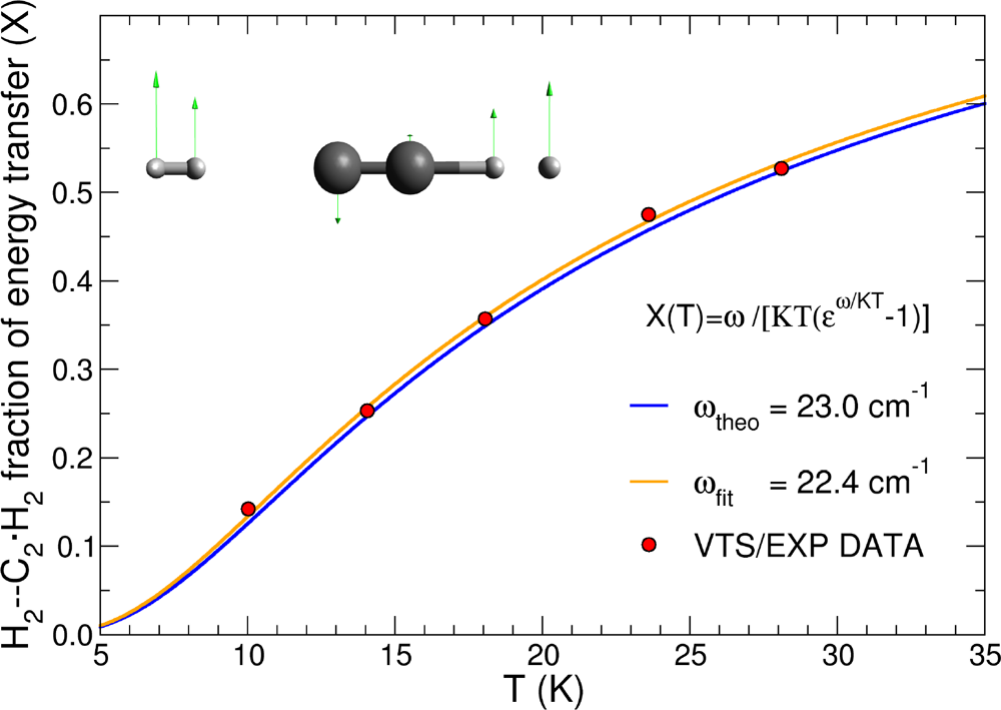
Numerical fit of the variation of the scaling parameter *x* as a function of temperature over the range of the five experimental points. The blue curve corresponds to a fit using the theoretically computed ω value, while the red curve uses the best fit of the points taken from the best agreement in Figure 4b between experiments and calculations. The inset depicts the internal bending motion of the 3B complex involving the anion partner and the two attached H_2_ molecules. The TS configuration is taken to be at the top of the barrier along the linear MEP. See the main text for further details.

Because of the fact that the scaling is dependent on the reaction temperature, we have first fitted the variations of *x* with *T*, as determined from Figure 6b, using a Bose–Einstein type of exponential form,^43^ where the computed frequency of the internal bending mode within the 3B complex was used as the value of ω for the fitting. This specific vibrational mode corresponds to an energy gap of 23 cm^–1^, and we shall discuss below its features. Since at *T* = 10 K the K*T* value corresponds to 6.95 cm^–1^, we see that the coupling can transfer more energy than that which would come from simple thermal interaction. By further employing eq 9, this procedure gave rise to the theoretical termolecular rate coefficients indicated by the blue curve in Figure 7. Next, we employed a numerical fitting procedure of the *x* values with a variable ω as a parameter and obtained the orange curve in Figure 7. It is reassuring to see that now the fitted ω value comes out to be fairly close to the theoretical value used by the blue curve. Such a test shows that the scaling values that best reproduce the experimental rate coefficients are also suggesting a physical mechanism for the 3B complex breakup via internal coupling with a specific vibrational mode. A pictorial view of the actual vibrational bending motion acting within the 3B complex and leading to its breakup into the products is shown in the inlet of Figure 7. It involves the three molecules in the reaction TS at the top of crossing the barrier and indicates an internal bending of the 2B complex under the coupling with the additional H_2_ molecule weakly bound to that complex. The second H_2_ molecule can also be detached from the complex either by further collisions with the buffer gas or by using the energy amount of about 180 meV, which is gained by the termolecular complex on the left of the barrier and going into the products region depicted in Figure 5.

These theoretical values are compared with the experimental quantities in Table 2. The values clearly show the good agreement between theory and experiment without any empirical scaling since the values of the fraction parameter *x* are obtained from our computed bending frequency within the TS termolecular complex and undergo no adjustment. Our present calculations thus indicate that the fraction of energy transferred by the second H_2_ molecule to the initial 2B complex chiefly flows via the specific internal bending motion just discussed.

**Table 2:** Comparison of the Experimental and Calculated Three-Body Reaction Rates at the Measured Temperatures^a^

Temperature (K)	Experiment	Calculations
10	11.4(7)	10.25
14	10.6(2)	9.47
18	9.3(2)	8.79
24	7.6(4)	6.32
28	5.8(2)	5.77

a The rates are given in units of 10^–30^ cm^6^/s.

## Conclusions

We performed temperature-dependent measurements of the reaction of C_2_^–^ with H_2_. Experimentally we find that the collision follows a pure quadratic dependence on the H_2_ density through a 3B reaction, leading to the charged C_2_H^–^ product. Hence, within experimental uncertainty, no bimolecular reactions were observed. The ternary reaction rate constants increase with decreasing temperature, and we find a temperature dependence of *T*^–0.82(12)^ and a rate constant of *a* = 8.2(3) × 10^–30^ cm^–6^/s at 20 K. This temperature dependence is in good agreement with theory using a classical approach on ion-atom-atom 3B recombination, which predicts a temperature dependence of *T*^–3/4^.

To elucidate the reaction process and to confirm the termolecular mechanism of this reaction, we carried out extensive *ab initio* calculations of the interaction forces between 2B and 3B complexes and further performed VTST rate coefficient evaluations, including tunneling, and considering both the 2B and 3B processes. The resulting rate coefficients turn out to be essentially negligible for the bimolecular reaction rates, while showing very good agreement with the experiments for the case where 3B collisions dominate the mechanism and where the second H_2_ acts as a chemical spectator assisting in the energy redistribution process by partially transferring the energy it gained before the barrier tunneling to the complex, thus leading to the formation of the C_2_H^–^ product. Our calculations found that the efficiency of this energy coupling step depends markedly on the temperature at which the reaction occurs, thereby indicating better energy transfer efficiency as the temperature in the trap increases.
